# Reviews

**Published:** 1880-01

**Authors:** 


### Reviews.

**A Ministry of Health and other Diseases**. By Benjamin Ward Richardson, M. D., F. R. S., &c New York: D. Appleton & Co. 1879.

Dr. Richardson includes in a volume of 354 pages, nine addresses: Ministry of Health; William Harvey; A Homily Clericc-Medical; Learning and Health; Vitality, Individual and National; The World of Physic; Burial, Embalming and Cremation; Registration of Diseases; Ether-Drinking and Extra-Alcoholic Intoxication.

In the address on Burial, Cremation and Embalming, the author, while expressing a preference for cremation, frankly confesses that "with all the fascinations of science which surround it, and with all the advantages which science lends for its development, it is simply impossible, as a general principle, at this imperfect stage of civilization." He contends, however, that the man of science should teach "that nature demands for the perpetuation of the living present, not the far removal, but the quick return of the dead body into the mother earth; that the world of life, constructed from a limited supply of matter, rebuilds itself out of the quarry of death, and that the plan which has for its object the restoration of the body to the earth, with the least possible interruption to the ordination of nature, should be accepted as the wisest plan."

The work is full of clear and forcible suggestions on subjects allied to sanitary science, and adds to the reputation of the author as an accomplished writer. L.

---

**The Pathology and Treatment of Venereal Diseases**. By FREEMAN J. BUMSTEAD, M. D. Tenth edition, revised, enlarged and in great part rewritten by the author and by ROBERT W. TAYLOR, M. D., Professor of Skin Diseases, in the University of Vermont. Philadelphia: Henry C. Lea.

Scarcely had this work appeared, when the sad intelligence reached us from New York, that the author, Dr. Freeman J. Bumstead, was dead. He died November 18th, after a protracted illness, but 53 years of age, being, at the time of his death, President of the New York County Medical Society. Thus a clear mind has passed away, and a valuable life is finished, but yet he will live in the memory of a grateful profession. His book is the best monument he could leave behind him.

The book itself is well known, but the new edition is so greatly enlarged and rewritten that it may be considered a new work.

It is naturally divided into three parts, Gonorrhœa and its complications (338 pages), the Chancroid and its complications (80 pages), and Syphilis (400 pages), giving a clear and concise statement of our present knowledge of venereal diseases. We earnestly advise the profession to buy this little book, not only on account of its general excellence and great clearness, but also as a fitting tribute to its late author. M.

---

**Infant Feeding, and its Influence on Life; or, the Causes and Prevention of Infant Mortality** By C. H. F. ROUTH, M. D., F. R. C. P. L. Third edition. New York: William Wood & Co. 1879.

We have given this work a careful and attentive perusal, and with a deep interest in the subject of which it treats, have come to endorse the wise judgment of the publishers in giving to the American profession the privilege of adding so valuable a contribution on infant dietics and therapeutics to their libraries. The author is evidently in thorough sympathy with his subject, and with the tender patients, for whose benefit he writes. He divides the work into three parts, of which the first is devoted to the consideration of the causes of mortality and viability of infants; the second to the subject of wet-nursing in its physiological as well as social relations; and the third to the general principles and practice of alimentation, while in the concluding portion of this valuable work, we find a clear and concise review of the symptoms and treatment, dietetic, hygienic and medicinal, of such diseases as tend to shorten life, or impair vital force through defective assimilation.

From this brief statement of the scope of the author's purpose in giving to the profession this valuable treatise, it will be seen that he has undertaken a work of the greatest importance to humanity. We feel that there prevails in the profession many crude notions on these subjects. The thorough study which is here given in a condensed form, suited to the general practitioner, will be of inestimable benefit, if only utilized and studied.

L.

**The Skin and its Troubles** New York: D. Appleton & Co., 549 and 557 Broadway. 1879.

This little work of 94 pages is one of the Health Primer Series, and treats, in a plain and popular manner, of the structure of the skin; the functions of the skin; practical applications to the conditions of daily life; skin troubles from poisonous clothing; the hair, and its ordinary management. It is replete with useful knowledge upon subjects of great interest and importance. We commend the work to the profession for distribution in their practice. L.

---

**First Line of Therapeutics as based on the Modes and Processes of Healing, as occurring Spontaneously in Disease, and on the Modes and Processes of Dying as Resulting Naturally from Diseases.** By ALEX. HARVEY, M. A., M. D., Emeritus, Prof. of Materia Medica in the University of Aberdeen, etc. New York: D. App'eton & Co.

While we have in the profession not a few, who fondly believe that drugs have a specific and curative power in the treatment of *all* diseases, it is well that the extremists, upon the other side of the question, who look upon the vis medicatrix naturæ as their only reliance, should have their say. Their views are in this book ably expressed, and notwithstanding its many sophisms, we venture to say that all who become possessors of the work will read it with pleasure and profit. Dr. Harvey takes the ground that in respect to diseases that are intrinsically curable, nature is herself adequate to the care of all of them, while in respect to those diseases, that are in their own nature incurable, art is also powerless. He denies wholly to drugs any specific curative powers. He says, page 277, "It is not we, it is not our drugs, that cure diseases; it is the organism itself which brings about spontaneously the restoration of health, through the manifold provisions, there are in the living body, for obviating the structural lesions induced by disease."

According to Dr. Harvey's notions, active therapeusis is a thing of the past, and "we may now have the inexpressible satisfaction, every one of us, of thinking that our practice is a partnership concern of the best kind—Nature doing the chief part of the work as regards the cure, but handing over to us the fees for the work done, and with the fees the credit of the cure."

D.

---

**The Throat and the Voice.** By J. SOLIS COHEN, M. D., Lecturer on Diseases of the Throat and Chest, in Jefferson Medical College, etc. Philadelphia: Lindsay & Blakiston.

Dr. Cohen is so well known that anything coming from his pen has a claim to our attention. In this little book, one of the health primers, he teaches the laity the common sources of diseases of the throat and voice and their symptoms, and gives general rules for their treatment. It is worth reading, and the advice it gives is sound. M.

---

**Diseases of Women.** By LAWSON TAIT, F. R. C. S. Second edition. New York: William Wood & Co. 1879.

**A Practical Manual of the Diseases of Children, with a Formulary.** By EDWARD ELLIS, M. D. Third edition. New York: William Wood & Co. 1879.

These works belong to "Wood's Medical Library Series," and maintain the deservedly high reputation of the publishers in their judgment of the wants of the profession in selecting useful and valuable medical books. They are concise, containing within a small compass a vast amount of professional erudition, which will be duly appreciated by the profession throughout the country. We have carefully examined this series, and with each number find it more and more adapted to the object the publishers have in view in presenting so many authorities on the different subjects of which they treat, at so small a cost. Without attempting a critical review of the works, we commend them heartily, and hope the enterprising publishers will continue to present standard authors in such a compact and uniform style and form. L.

